# Metal leaching from antimicrobial cloth face masks intended to slow the spread of COVID-19

**DOI:** 10.1038/s41598-021-98577-6

**Published:** 2021-09-28

**Authors:** Zoe A. Pollard, Madeline Karod, Jillian L. Goldfarb

**Affiliations:** Department of Biological & Environmental Engineering, Cornell University, Ithaca, NY 14853 USA

**Keywords:** Environmental chemistry, Chemical engineering, Materials chemistry

## Abstract

Global health organizations recommend the use of cloth face coverings to slow the spread of COVID-19. Seemingly overnight, companies whose primary business is in no way related to healthcare or personal protective equipment—from mattresses manufacturers to big box stores—transitioned into the “mask business.” Many companies advertise antimicrobial masks containing silver, copper, or other antimicrobials. Often, the techniques used to load such antimicrobials onto mask fibers are undisclosed, and the potential for metal leaching from these masks is yet unknown. We exposed nine so-called “antimicrobial” face masks (and one 100% cotton control mask) to deionized water, laundry detergent, and artificial saliva to quantify the leachable silver and copper that may occur during mask washing and wearing. Leaching varied widely across manufacturer, metal, and leaching solution, but in some cases was as high as 100% of the metals contained in the as-received mask after 1 h of exposure.

## Introduction

One of the most politicized and now commercialized aspect of the COVID-19 pandemic is the use of cloth face coverings among the general public to mitigate the spread of SARS-CoV-2^1^. Decades of research demonstrate the effectiveness of medical-grade surgical and N-95 masks against aerosolized disease transmission^2–4^. A new corpus overwhelming agrees that even cloth face masks provide some protection against infection of the wearer and effectively reduce community spread of SARS-CoV-2^5–7^. As the US death toll from COVID-19 mounts, with cases surging above 36.5 million (as of 12 August 2021), mask-wearing was recently called one’s “civic duty, a small sacrifice reliant on a highly effective low-tech solution that can help turn the tide favorably in national and global efforts against COVID-19^8^.” While the precise level of effectiveness of cloth face masks varies by fabric, number of layers, and fit, fabric masks may filter out greater than 95% of the virus present in aerosols > 5 µm^9^, with tight-weave fabric blends capable of removing up to 80% of particles smaller than 300 nm in size^10^.

Global health guidelines to wear a cloth face covering were (initially) motivated by limiting community spread^11^, especially by asymptomatic infected persons^12^. However the increased use of cloth face masks by the US population (from roughly 62% of people in April to 76% in May, 2020) is strongly attributed to the belief that mask-wearing reduces one’s own risk of infection^13^. On 27 July 2021, the US Centers for Disease Control and Prevention (CDC) reversed its recommendations, which previously (with guidance issued on 18 May 2021) exempted vaccinated individuals from mask-wearing in public, in light of the highly contagious Delta variant. At publication, the current CDC recommendation is that even vaccinated individuals should wear masks indoors when the risk of transmission is high or substantial, including in schools^14^. This has perhaps fueled the commercialization of masks with “enhanced” features, such as antimicrobial properties. There are now hundreds of retailers selling antimicrobial cloth face masks across the internet from biomedical fiber companies to clothing retailers to mattress makers. Some companies, such as Sonovia Ltd., claim to have scientific testing that proves their mask—impregnated with zinc oxide nanoparticles—is over 90% effective at killing SARS-CoV-2, even after 100 washings^15^. The majority of companies selling so-called antimicrobial masks add disclaimers to their products, such as “This product makes no claims that it will cure or prevent Corona Virus, Influenza, or Covid-19^16^.” In a recent NBC news story, Jeffrey Keane, CEO of Noble Biomaterials, claimed that antimicrobial masks help reduce the risk of virus transmission by eliminating cross contamination from fabric to skin^17^. This optimism is tempered by comments from medical experts suggesting such claims are over-inflated and may imbibe the wearer with a false sense of protection^17^. Regardless of the effectiveness of antimicrobial masks against SARS-CoV-2, metal-infused face masks may pose a potential risk to wearers in the form of metal leaching.

Many antimicrobial textiles are metal impregnated, often with copper or silver engineered nanoparticles (ENPs)^18–25^. These ENPs provide antimicrobial benefits by releasing metallic ions (Ag^+^ or Cu^2+^) or reactive oxygen species which can damage the bacterial wall in an aqueous environment^26^. However, once loosened from the fabrics, these metal species could leach into graywater (via washing) or onto the consumer’s skin during wearing. In one of the first studies to demonstrate the potential leaching behavior of commercially available ENP-impregnated textiles, Benn and Westerhoff observed up to 650 µg of silver leached into 500 mL of distilled water from a single commercial sock^27^. Washing and rinsing of silver-impregnated textiles can release everything from coarse particulate matter (45 µm and larger), to nanosilver, to dissolved silver^28^. Detergents, basic in nature (pH > 10) with a variety of complexing agents and ions, can interact with silver present, leading to AgCl and Ag_2_S formation^28,29^. Bleaching agents can oxidize nano-Ag and lead to dissolution^30^ and AgCl formation^31^. Similar results are noted for leaching of copper from Cu-ENP-impregnated textiles^32^. Overall, the release and transformation of ENPs from antimicrobial textiles is mediated by (1) initial metal incorporation into the fibers^32^ (location, adhesion method, textile composition, ENP/metal salt form); (2) textile application (body temperature, sweat level, abrasion/activity); (3) washing/cleaning (modality and chemicals); (4) disposal^33^.

To our knowledge, there are no studies (to date) that assess the potential for saliva to promote metal leaching from textiles, likely owing to the previously limited use of face coverings pre-COVID-19. Oral ingestion, inhalation, and dermal adsorption of leached metals from wearing a copper- or silver-infused face mask are potential metal exposure pathways. Studies from the dental literature show the potential for decomposition of, and metal leaching from, metal alloys used in dental implants due to the antioxidant properties of saliva^34^. This led us to hypothesize that saliva could promote the leaching of silver and/or copper from a fiber matrix. While silver exhibits relatively low toxicity as compared to other metals, ingestion and dermal adsorption can result in argyria and argyrosis (irreversible skin and eye pigmentation)^35^ at low concentrations. Generalized argyria can result from inhalation and application of silver to mucosal membranes, which has led to seizures and organ damage^36^. At higher concentrations, soluble silver inhalation can cause respiratory irritation and reductions in glutathione, critical for red blood cell functions^37^. Copper, while being an essential micronutrient, can cause gastrointestinal issues with chronic adult exposure at doses above 5.3 mg^38^. Exposure to copper dust can cause respiratory irritation, headache, vertigo, and hepatomegaly^39^. As such, the present study examines the potential for leaching of silver and copper from anti-microbial face masks due to exposure to saliva and during simulated wash/rinse cycles to shed light on both human and environmental health implications. For this study, 9 masks with advertised antimicrobial benefits (and one without any advertised antimicrobial properties as a control) were selected from varying manufacturers and the potential for metal leaching from each mask was investigated. Mask descriptions are provided in Table 1.

**Table 1:** Commercially available “antimicrobial” masks analyzed (for fiber composition, “inner” refers to fabric touching the mouth, “outer” to the fabric facing outwards, “fill” to any lining material between the inner and outer fabric).

Mask number	Primary industry of manufacturer	Fiber composition	Product labeling re: antimicrobial properties	Manufacturer’s washing notes	Mass of as-received mask (g) and (size)
1	Mattresses	2-layer cotton with polyester fill	Copper infused threads	Washable	15.81 (large)
2	Sportswear	3-layer cotton	Contains silver and copper	Machine washable up to 15×	14.58 (large)
3	Clothing	2-layer polyester with cotton fill	Anti-microbial fabric; 60% cationic, 40% polyester	Washable	21.37 (M/L)
4	Shoes	Inner: Cu-lined ionized quartz yarn fabric, outer: polyester	Anti-microbial copper	Washable up to 50×	11.96 (large)
5	Medical clothing	Inner: polyester, outer: 84% polyester, 11% rayon, 5% spandex	Treated with Silvadur™	Washable	15.46 (one size)
6	Medical clothing	Inner: cotton, outer: 97% cotton, 3% spandex	Treated with Silvadur™	Washable	12.73 (one size)
7	Clothing	2-layer 65% polyester, 35% rayon with polyester fill	Anti-microbial coating	Washable up to 30×	15.44 (large)
8	Materials consulting	2-layer polyester	Antimicrobial SilverKiss™	Washable	20.02 (L/XL)
9	Athletic accessories	2-layer cotton with 1 layer polypropylene fill and 1 layer polyester fill	Copper infused	Washable	12.80 (one size)
10 (control)	General merchandise	2-layer cotton	None	Washable	11.78 (L/XL)

## Results and discussion

Initial silver and/or copper loading in each as-received mask was measured via Inductively Coupled Plasma - Mass Spectrometry (ICP-MS) (Table 2); at least two masks per manufacturer and one sample per mask were used to ensure a representative sample. Silver is present in Masks 2, 4, 5 (up to 2 mg per mask with masks weighing between 11.96 and 15.46 g), and at considerably higher concentrations in Mask 8 (upwards of 100 mg per mask with a total mask mass of 20.02 g). Copper was detected in Masks 1, 2, 4, 6 and 9 at levels between 2 and 14 mg per mask. We note that while Mask 6 was advertised as being treated with DuPont’s Silvadur™, multiple repeated experiments across three separate masks from the same manufacturer detected no silver (yet found significant copper) upon complete digestion of the mask. In addition, Mask 4 was advertised as containing “copper impregnated threads,” though silver was also detected. Masks 3 and 7 are advertised as being made from an antimicrobial fabric, but as they contain no (detectable) silver or copper; antimicrobial properties are likely imparted by a quaternary ammonia compound coating. As such, Masks 3 and 7 are excluded from the remaining discussion in terms of potential Ag and Cu leaching. It is important to note that since the nature of the antimicrobial coating is often not advertised (or, as we have found, mis-advertised) there is potential for additional health hazards resulting from the exposure to other chemicals used. Only Cu and Ag leaching are investigated in this work. As expected, Mask 10 (Control) contains no detectable silver or copper, demonstrating the validity of the analysis method with a null finding. The high reproducibility of metal concentrations across at least two samples per mask analyzed at least thrice each (evidenced by the tight 95% confidence intervals around each data point) and our continuous ICP-MS blank and calibration checks (between every 10 samples analyzed) suggest minimal ICP-MS carry-over and no cross-contamination of samples.

**Table 2:** Total silver and/or copper present in as received masks; n.d. denotes samples where metal was not detected.

Mask	Metal present in mask (mg_metal_/g_mask_)									
	1	2	3	4	5	6	7	8	9	10
Silver	n.d.	0.02	n.d.	0.16	0.03	n.d.	n.d.	5.27	n.d.	n.d.
Copper	0.80	0.09	n.d.	1.11	n.d	0.11	n.d.	n.d.	0.65	n.d.

Standard deviation for every value is less than 0.01 mg metal/g mask.

**Figure 1:**
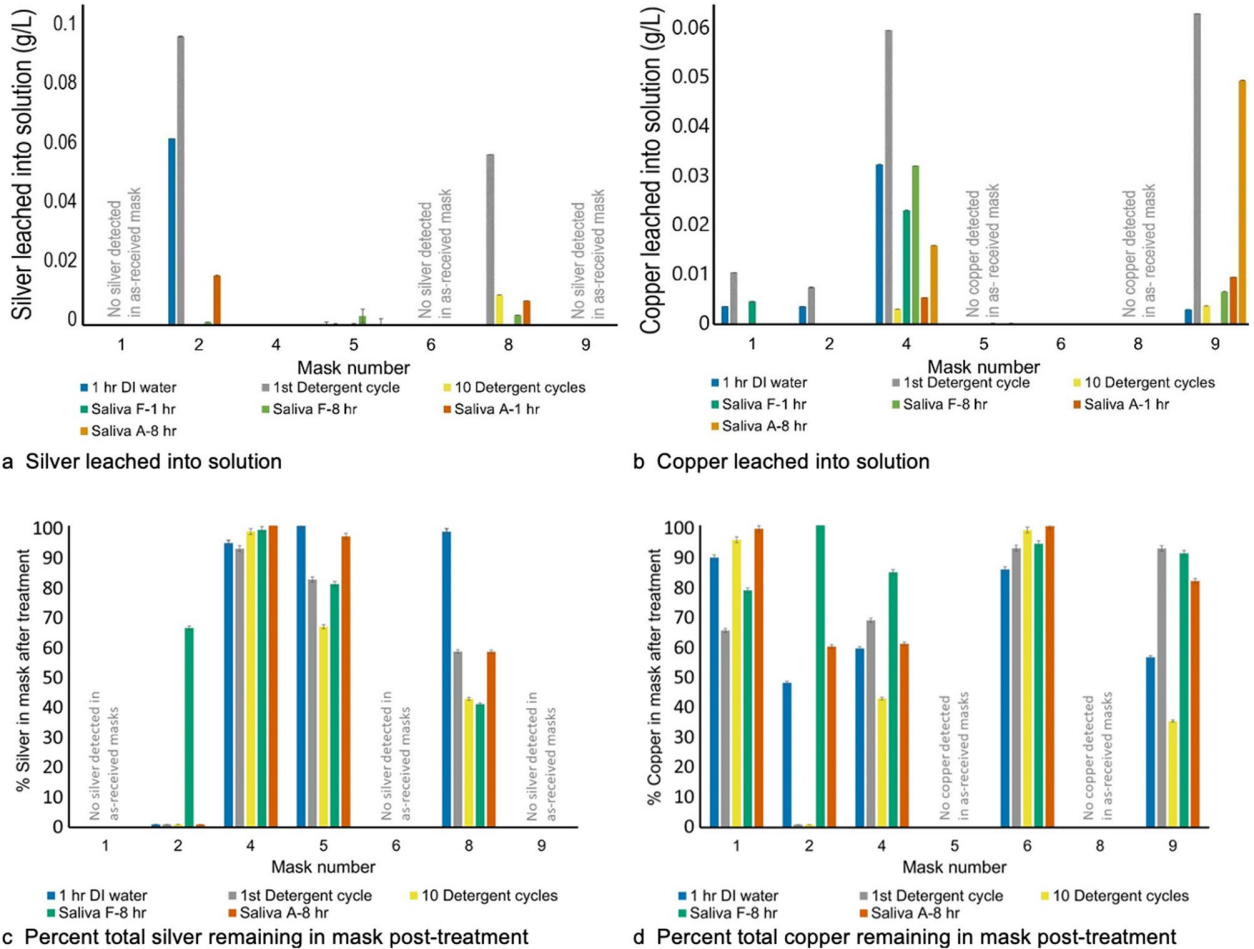
Total silver and/or copper leached into treatment solution (**a**,**b**) and present in masks post-treatment (**c**,**d**); error bars represent ± one standard deviation about the mean.

Figure 1 presents the leaching of silver (1a) and copper (1b) into treatment solutions and the percentage of silver (1c) and copper (1d) remaining in masks post water rinse, simulated washing, and saliva exposure. Only data for masks initially containing metal (1, 2, 4, 5, 6, 8, 9) are shown here, though all masks were subjected to every test (tabulated values for all experiments available in Supplemental Information Table S1 (silver) and Table S2 (copper), available online). Figure 1a,b show the quantity of silver and/or copper that has the potential to leach from an antimicrobial mask into a given solution. A high degree of leaching for both metals was observed in a single wash detergent cycle, indicating the potential for graywater contamination because of washing the antimicrobial masks. It is important to note that after 10 detergent cycles the leaching levels in solution (Fig. 1a,b) are considerably lower than after 1 cycle. This is not because the metals were stabilized by the detergent (as after 10 washes there is considerably less metal remaining in the mask), but rather that metals were leached by the detergent in the previous cycles (wash water from wash cycles 2–9 was not analyzed due to experimental constraints). The ratio of the original metal remaining after treatment is shown in Fig. 1c,d and was determined by digesting and analyzing the masks as received and after treatment. A mass balance on each metal was performed by summing the metal present in the soak solution and the residual metal present in the mass, divided by the metal detected in the as received mask. The majority of the samples had mass balance closure within ± 10% of the as received mask data. Given well-documented potential heterogeneities of metal application in the mask fibers themselves^40^, this high mass closure validates our experimental methods. We note that the mass closures for the single detergent cycle are highly variable (between 31 and 106%), due likely to both metal distribution heterogeneity *and* the rinse cycles used. While we attempted to analyze the metals present in the water rinse following washing, the metals were below limits of detection due to the rinse volume.

Overall, we note that the total amount of metals leached does *not* correspond to the amount initially present, suggesting that initial metal concentration is not driving partitioning to the solution phase. For example, Mask 2 has a relatively low metal loading (0.236 ± 0.003 mg_Ag_/mask + 1.29 ± 0.04 mg_Cu_/mask), yet the silver appears to leach almost entirely after just one wash/rinse cycle, and extensively in both water and Saliva A. (No silver was detected in the 10th wash cycle solution as the silver was completely removed by the first wash cycle.) While water alone was sufficient to remove the majority of Ag from this mask, it did not have the same impact on the Cu present. Only 52% of the copper leached from Mask 2 during the 1-h water soak, but 100% was lost after 1 wash cycle. No leaching was detected in the 10th wash cycle for either metal, as all the metals leached from Mask 2 in the first cycle. As detergents are intended to remove foreign matter from fabric, it is not surprising that the detergents washes show, on average, the largest amount of leaching for both metals and across all masks. The complexing agents present in detergents were previously demonstrated to interact with silver metals present in textiles, leading to AgCl formation and dissolution^28,29^.

As compared to Mask 2, Mask 8 has a considerably higher silver loading (105.4 ± 0.5 mg_Ag_/mask), but rather low silver leaching, retaining all its silver following the DI water soak and close to 50% of silver following the other treatments. Mask 6 (also with low loading of 1.44 ± 0.04 mg_Cu_/mask) has a constant copper concentration across the treatments, suggesting its potential to leach metals either onto the wearer’s saliva or during washing is minimal. This suggests that the metals are more tightly bound to the textile of the mask. As such, neither detergents nor the acidic and oxidizing nature of the saliva solution removed them from the fiber matrix.

Given that the differences in the amount of metal leaching during washing cannot be attributed to initial concentration, they may be due to the method of metal loading and the fiber content of the mask. Reed et al*.* found that textiles coated with elemental silver released less than 2% of loaded silver in both DI water and detergent, whereas tethered-AgNP and electrostatic Ag-NP attachments resulted in more than three quarters of Ag released during DI washing and half during detergent washing. Silver salt-coated textiles were between these, with about 20% silver released during DI water and detergent wash cycles^41^. Pasricha et al*.* synthesized AgNPs via solution reduction and physically loaded the particles onto three fabrics. The cotton fiber, having a 31% initial loading, leached 12% of the silver after three washes, nylon fibers with 11% initial Ag loading leached 14% of the silver after three washes, whereas wool, with an initial 10% Ag loading, leached over one quarter of the silver^42^. From these and other examples, we know that metal leaching is a function of fiber content, loading method and leaching solution. The masks examined in this study exhibit a wide range of metal leaching behavior. Mask 4 maintains a high percentage (> 90%) of its initial silver across all treatments, however it leaches between 20 and 60% of the copper initially present.

In general, copper appears more susceptible to leaching when at higher concentrations than the silver across treatments. Masks 6 and 9 both contained copper. Mask 6 is predominantly cotton fiber, whereas Mask 9 is a blend of cotton, polypropylene, and polyester. Mask 9 leached significant amounts of copper after 10 washes (approximately 65% of its initial concentration) and into Saliva A (almost 20%) in contrast to Mask 6 which exhibited minimal leaching in any aqueous environment. In addition to the variability in fiber type (both loading metal and fiber content), this leaching behavior could be attributed the incorporation method of the metal. Mask 6 is advertised to be treated with a polymer-based coating, while the fibers of Mask 9 were ‘infused’ with copper.

Saliva A is reported to be more aggressive in terms of its oxidation properties^43^, but its impact here versus Saliva F is mask- and metal-dependent. Saliva A leaches more Ag than Saliva F for Masks 2 and 4 (which contains both Ag and Cu) but leaches less Cu for either mask than Saliva F. Masks 5 and 8 (containing only Ag) and Mask 1 (containing only Cu) exhibit less leaching in Saliva A than in Saliva F. This may be attributed to the lower pH of Saliva F; the acidity of the saliva solutions may stabilize the silver. Mask 9 leaches between 10 and 20% of its copper into artificial saliva A and F, respectively, after 8 h, suggesting that over the course of a workday. The greatest risk from copper leaching appears to be posed by Mask 4 and 9, and from Ag leaching from Mask 8, especially if it is not pre-washed.

The degree of toxicity of silver is dependent on its oxidation state and morphology; silver nanoparticles (AgNPs) are less toxic than silver ions^44^. However, the moist environment of a face mask and the known rapid dissolution of AgNPs to Ag^45^ suggests that the total Ag measurement in this study is representative of the most toxic potential silver exposure possible as the metals are in a dissolved form. These conditions are representative of what many essential workers endure, including preparing food in hot kitchen, waiting tables, working construction, and hauling goods, where it is likely for masks to become saturated over the course of a workday^46^. Studies have shown that in humid environments, or when worn for long periods of time, masks quickly become saturated^47^ with saliva droplets^48,49^ that are expelled during talking, sneezing, or coughing^50^. In the most extreme cases, if we assume an average adult weighs 70 kg, the potential exposure^51^ (over an 8 h. workday, simulated saliva) to silver could be as high as 900 µg/kg (Mask 8) and to copper as high as 75 µg/kg (Mask 4). To further explore this, UV–Vis spectrophotometry was used as a screen for metal nanoparticles in the treatment solutions. The lack of any significant UV–Vis absorption bands in the 400–460 nm range, which would indicate surface plasmon resonance of AgNPs, confirm that the Ag is in dissolved form^52^ (see Supplementary Figures S1 and S2 online). Spectrophotometry results are less conclusive concerning the form of Cu leached; Cu nanostructures tend to show broad and weak localized surface plasmon resonance responses, though the lack of peaks above 500 nm suggest the copper is more likely in a dissolved form^53^. However, some evidence of red shifts for Masks 1, 2 and 9 for Saliva F and detergent solution-based samples cannot rule out nano-Cu forms completely^54^, especially for Mask 2 that shows peaks at 275 and 375 nm for several leaching solutions^55^.

Public calls to temper the enthusiasm over antimicrobial masks (and their unlikely ability to protect oneself against COVID-19 infection beyond non-enhanced cloth masks) are supported by the likelihood that wearers are exposed to metals leaching from the mask fibers. For masks produced by a shoe manufacturer (Mask 4) and a materials consulting firm (Mask 8), this leaching is promoted in the present of saliva, providing a direct exposure route for the wearer. A series of 10 masks, 7 of which contained copper and/or silver as antimicrobial agents, were exposed to deionized water, commercial laundry detergent (1 and 10 simulated washing cycles), and two artificial saliva solutions. While some masks showed minimal loss of metals, others, such as one manufactured by a sportswear company (Mask 2), leached 100% of its silver after exposure to deionized water, detergent, and Anfor artificial saliva solution and 35% after exposure to Fusayama artificial saliva solution. This exposure risk could be reduced by first washing the masks, however metal leaching in detergent solutions likely reduces any antimicrobial activity and leads to graywater contamination. Overall, the results of this investigation suggest that metal-impregnated antimicrobial masks have the potential to contaminate wastewater streams and increase human exposure to silver and copper, which may pose a health risk at even the low concentrations detected here. While manufacturers rapidly shifted production lines to meet the demand for masks at the onset of the pandemic, the present investigation suggests that not all so-called antimicrobial masks are equal, with some posing a greater threat to humans and the environment than others.

## Materials and methods

Masks labeled as “antimicrobial” were purchased from a variety of internet retailers in July 2020 as detailed in Table 1. A set of control “plain” cloth masks with no antimicrobial features was purchased at a local “big box” retail store where over 50% of Americans shop nationwide^56^. Masks were cut with new fabric shears into 2 cm × 2 cm ^2^ through all layers of the masks with straps and seams excluded.

### Simulated product lifecycle exposure via washing

The CDC recommends daily laundering of cloth face masks by including the mask with one’s “regular laundry” at the “warmest appropriate water setting for the cloth used to make the mask”^57^. Tide Original HE Turbo Clean was chosen as a representative laundry detergent as it is the best-selling laundry detergent in the US for over 70 years^58^. Detergent washing solution was prepared by diluting 20 mL Tide in with 980 mL Milli-Q water. To simulate washing, a 2 cm^2^ sample of each mask was soaked in 6 mL of detergent solution at 37 °C while shaking at 100 rpm for 1 h in an Innova 44R Incubator Shaker (Eppendorf New Brunswick). Samples were rinsed in 10 mL of Milli-Q water, at 37 °C with shaking at 100 rpm for 1 h. For repeated washing cycles the wash and rinse were repeated ten times with the rinse and soak solutions analyzed for the first wash and the tenth wash.

### Simulated product lifecycle exposure via wearing

Prior studies on metal leaching from textiles consider an article of clothing worn on the body, where sweat and abrasion would be the most common causes for metal release. In the case of a face mask covering the mouth, the environment is warm air/CO_2_, humidified by saliva, a fluid with known antioxidant properties. Fusayama artificial saliva solution (denoted saliva F; pH = 5)^59^ and Anfor saliva solution (denoted saliva A; pH = 6.7)^60^ were used as a simulated leaching fluid for as-received masks. Artificial saliva solutions were mixed in house by dissolving and vortexing the following compounds in Milli-Q water; saliva F contained NaCl (0.4 g/L), KCl (0.9 g/L), CaCl_2_⋅2H_2_O (0.795 g/L), NaH_2_PO_4_ (0.69 g/L), and urea (1 g/L), and saliva A contained NaCl (0.7 g/L), KCl (1.2 g/L), Na_2_HPO_4_ (0.26 g/L), NaHCO_3_ (1.5 g/L), KSCN (0.33 g/L), and urea (1 g/L). All compounds had a minimum purity of 99.0% (by mass) with a certified metal analysis reporting no detectable copper or silver. While Fusayama solution is the most used media in the evaluation of dental implants, the composition of human saliva is known to vary widely. Thus, two saliva solutions were used for the mask leaching experiments with the calcium component in Fusayama tempering the oxidant nature of the more pro-oxidant Anfor solution^43^. 2 cm square mask samples were soaked in 10 mL of each artificial saliva solution for 8 h to simulate a typical workday.

### Analysis of total metal content and leached metals

DI water soak, wash/rinse, and artificial saliva soak solutions were analyzed (undiluted) for the presence of metal nanoparticles using UV–Vis spectrophotometry. Measurements were carried out using a Shimadzu UV–Vis Spectrophotometer in absorbance mode. Spectra were recorded across a wavelength range of 250 nm to 600 nm with a 1 cm sample depth and a 1 nm spectrometer slit width.

To prepare the mask samples for ICP-MS analysis, 0.01 g of as-received and treated masks (after 1 h DI water, detergent wash cycles, and artificial saliva exposure) were digested in 2 mL 70% trace metal grade nitric acid at 70 °C for 16 h, centrifuged, and the resulting supernatant extracted and filtered through a 20 μm hydrophilic cellulose acetate syringe filter. Aliquots of the soaking solutions were mixed 1:1 with 70% trace metal grade nitric acid at 65 °C for 2 h then centrifuged and the resulting supernatant extracted. Prior to ICP-MS analysis all digested samples were diluted to 2% nitric acid using Milli-Q water. Diluted solutions were analyzed for metal concentration using a Shimadzu Inductively Coupled Plasma Mass Spectrometry (ICP-MS-2030). Analysis was done in quantitative mode using an internal standard and a 9-point calibration curve. The tuning solution, calibration solution and internal standard were all purchased from High Purity Standards (USA). Additional ICP-MS details are available in the online Supplemental Information.

### Statistical analysis

All mask-soaking experiments were run in (at least) duplicate and analyzed independently. The Shimadzu UV–Vis Spectrophotometer and Shimadzu Inductively Coupled Plasma Mass Spectrometer analyze samples in triplicate. The means and standard deviations are reported of the triplicate measurements of duplicate runs.

## Supplementary Information

Supplementary Information.

## Data Availability

The masks used in this study are available for commercial purchase. All data used in this paper has been included in the Supplemental Information in graphical and/or tabular form.
