# Supplementary material for: Metal leaching from antimicrobial cloth face masks intended to slow the spread of COVID-19
Source: Sci Rep. 2021 Sep 28;11:19216. doi: 10.1038/s41598-021-98577-6 (PMC8479130; doi:10.1038/s41598-021-98577-6)
Supplement: Supplementary file 1 — Supplementary Information. [file 41598_2021_98577_MOESM1_ESM.docx]

**Metal Leaching from Antimicrobial Cloth Face Masks Intended to Slow the Spread of COVID-19**

Zoe A. Pollard, Madeline Karod, Jillian L. Goldfarb^[[1]](#footnote-1)^*

Department of Biological & Environmental Engineering, Cornell University, Ithaca NY 14853, USA

**Supplemental Information** (for online publication only)

Contents

| **Table S1** | Silver concentration as measured by ICP-MS ± one standard deviation about the mean | S1 |
| --- | --- | --- |
| **Table S2** | Copper concentration as measured by ICP-MS ± one standard deviation about the mean. | S2 |
| **Table S3** | Mass balance closures on metals recovered from masks and soak solutions | S3 |
| **Figure S1** | UV-Vis spectra of as-used (pure) soak solutions including diluted detergent for wash cycles, DI water for rinse, and both artificial saliva solutions. | S4 |
| **Figure S2** | UV-Vis spectra of solutions post mask-exposure sorted by treatment | S5 |
| **Appendix S1** | Supplemental ICP-MS experimental details | S6 |

**Table S1.** Silver concentration as measured by ICP-MS ± one standard deviation about the mean.

**Table S2:** Copper concentration as measured by ICP-MS ± one standard deviation about the mean.

**Table S3.** Mass balance closures on metals recovered from masks and soak solutions

Notes: Calculations for 10 detergent cycle not performed as samples from washes 2-9 were not analyzed. Recoveries for 1 detergent cycle are (often) low as only detergent solution was analyzed (rinse water to prepare mask for ICP-MS digestion not analyzed as volume of water used made metals below limit of detection)

**
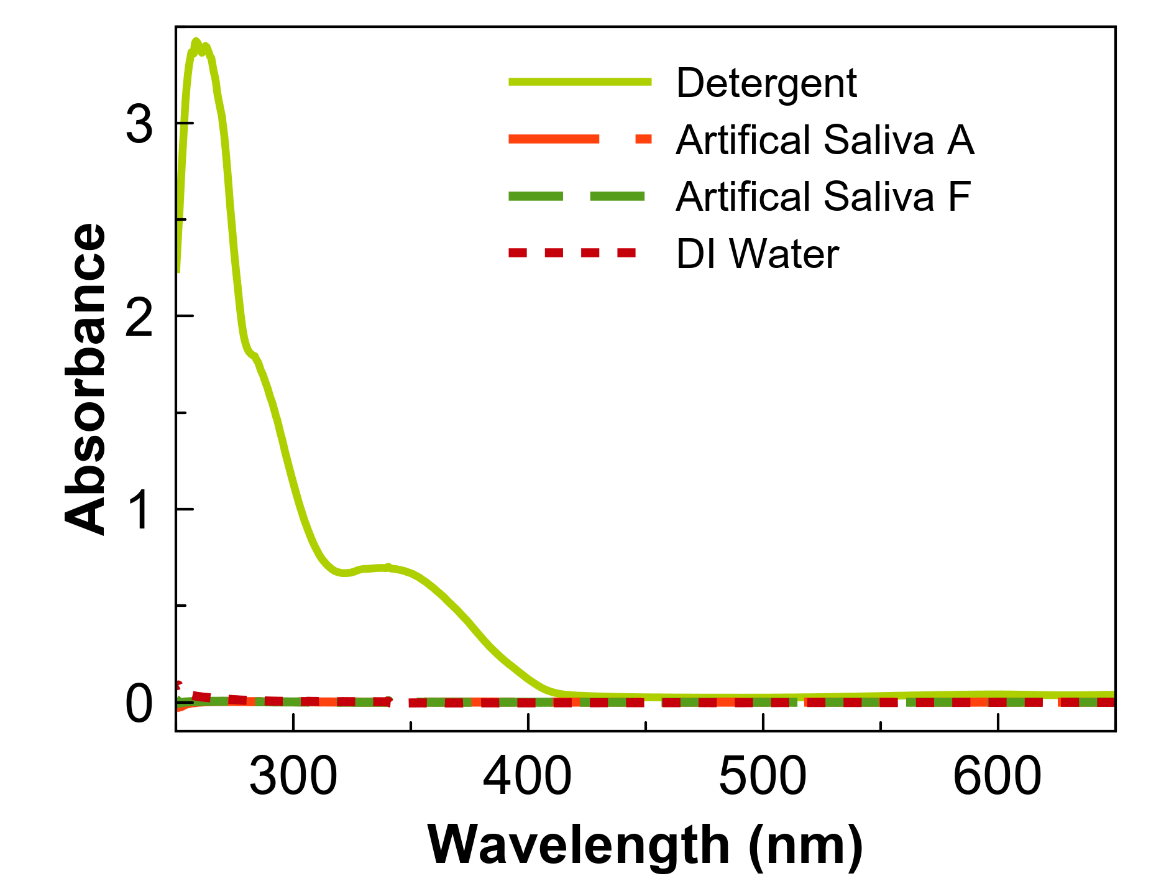
**

**Figure S1.** UV-Vis spectra of as-used (pure) soak solutions including diluted detergent for wash cycles, DI water for rinse, and both artificial saliva solutions.

| 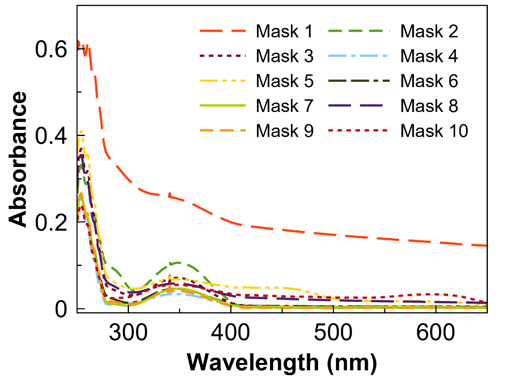 | 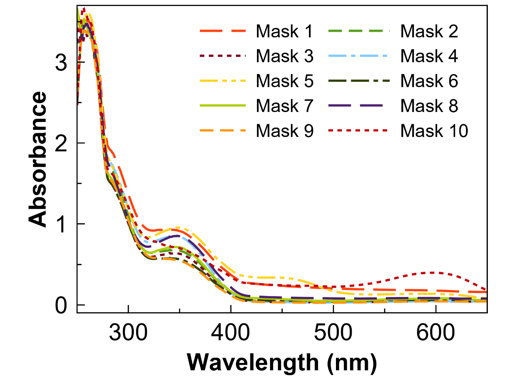 |
| --- | --- |
| a. DI water soak | b. 1st Detergent cycle soak |
| 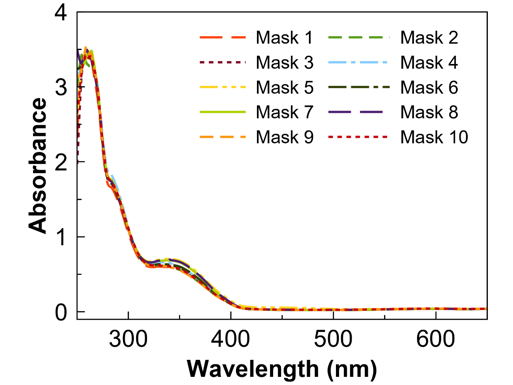 | 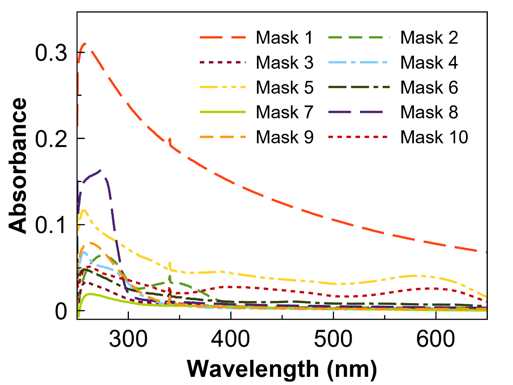 |
| c. 10th Detergent cycles soak | d. Artificial Saliva A soak before washing |
| 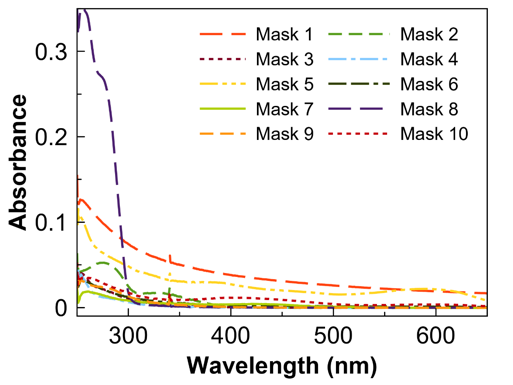 | 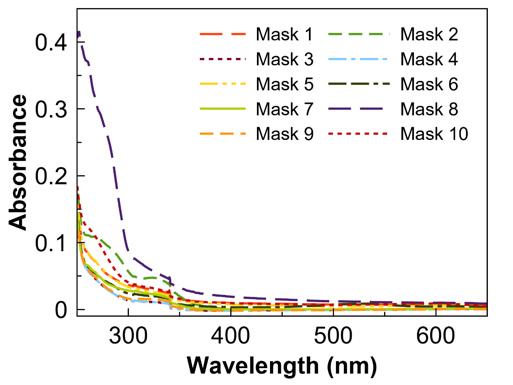 |
| e. Artificial Saliva F soak before washing | f. Artificial Saliva F soak after 1^st^ wash cycle |
| 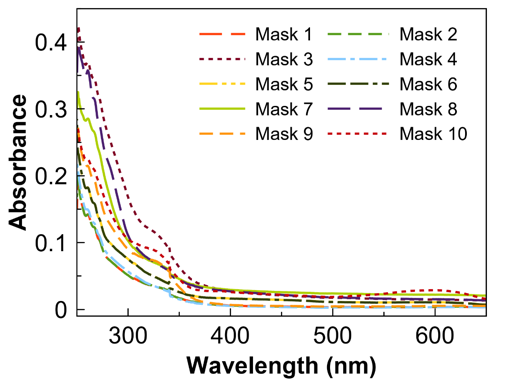 |  |
| g. Artificial Saliva F soak after 10^th^ wash cycle |  |

**Figure S2.** UV-Vis spectra of solutions post mask-exposure sorted by treatment.

**Appendix S1: Supplemental ICP-MS experimental details**

The Shimadzu ICP-MS-2030 is equipped with a mini-torch plasma torch, a coaxial nebulizer, 27 MHz high-frequency power supply unit, and a quadrupole mass spectrometer. The instrument was validated and aligned with tuning solution (High Purity Standards, IV-STOCK-71). The argon carrier gas (Airgas, Certified Ultra-High Purity Argon) was controlled using a Matheson regulator to an inlet pressure of 450 kPa as recommended by the instrument manufacturer. The He collision cell was not utilized as a nine-point linear calibration curve in the range of 2-2000 ppb with an R-value >0.9999 was achieved without the introduction of helium gas.

Metal concentrations below 2 ppb are below the limit of detection for the instrument and would correspond to a maximum silver and/or copper content of 0.05 mg/g_Mask_. Samples were analyzed for concentrations of silver (mass 107) and copper (mass 63) with an internal standard (High Purity Standards, ICP-MS-IS-1) containing 10 µg/mL Bi, Ho, In, ^6^Li, Lu, Rh, Sc, Tb, and Y in 2% HNO_3_ + Tr HCl. All samples were diluted with ultra-pure trace metal grade nitric acid and Milli-Q water. Blank solutions using the same lot of nitric acid and Milli-Q water were run between every 10 samples, and metal concentrations were standardized to the blank solutions to eliminate the chance of background metal detection.

All disposable plasticware was soaked in a 10% trace metal grade acid bath for 24 hours then rinsed with Milli-Q water and dried, covered. This was done to ensure that no external contamination was present.

1. * To whom correspondence should be addressed: goldfarb@cornell.edu; JillianLGoldfarb@gmail.com; 607.255.5789 [↑](#footnote-ref-1)
